# Estimates for quality of life loss due to Respiratory Syncytial Virus

**DOI:** 10.1111/irv.12686

**Published:** 2019-10-18

**Authors:** David Hodgson, Katherine E. Atkins, Marc Baguelin, Jasmina Panovska‐Griffiths, Dominic Thorrington, Albert Jan van Hoek, Hongxin Zhao, Ellen Fragaszy, Andrew C. Hayward, Richard Pebody

**Affiliations:** ^1^ Centre for Mathematics, Physics and Engineering in the Life Sciences and Experimental Biology University College London London UK; ^2^ Department of Mathematics University College London London UK; ^3^ Centre for the Mathematical Modelling of Infectious Diseases London School of Hygiene & Tropical Medicine London UK; ^4^ Department of Infectious Disease Epidemiology Faculty of Epidemiology and Population Health London School of Hygiene & Tropical Medicine London UK; ^5^ Centre for Global Health Usher Institute of Population Health Sciences and Informatics Edinburgh Medical School The University of Edinburgh Edinburgh UK; ^6^ Respiratory Diseases Department Public Health England London UK; ^7^ Department of Applied Health Research University College London London UK; ^8^ Department of Epidemiology and Surveillance National Institute for Public Health and Environment Bilthoven The Netherlands; ^9^ Centre for Public Health Data Science Institute of Health Informatics University College London London UK; ^10^ Department of Epidemiology and Public Health University College London London UK

**Keywords:** cost‐effectiveness, EQ‐5D, health‐related quality of life, human respiratory syncytial virus, quality‐adjusted life years, respiratory disease

## Abstract

**Background:**

In children aged <5 years in whom severe respiratory syncytial virus (RSV) episodes predominantly occur, there are currently no appropriate standardised instruments to estimate quality of life years (QALY) loss.

**Objectives:**

We estimated the age‐specific QALY loss due to RSV by developing a regression model which predicts the QALY loss without the use of standardised instruments.

**Methods:**

We conducted a surveillance study which targeted confirmed RSV episodes in children aged <5 years (confirmed cases) and their household members who experienced symptoms of RSV during the same time (suspected cases). All participants were asked to complete questions regarding their health during the infection, with the suspected cases additionally providing health‐related quality of life (HR‐QoL) loss estimates by completing EQ‐5D‐3L‐Y or EQ‐5D‐3L instruments. We used the responses from the suspected cases to calibrate a regression model which estimates the HR‐QoL and QALY loss due to infection.

**Findings:**

For confirmed RSV cases in children under 5 years of age who sought health care, our model predicted a QALY loss per RSV episode of 3.823 × 10^−3^ (95% CI 0.492‐12.766 × 10^−3^), compared with 3.024 × 10^−3^ (95% CI 0.329‐10.098 × 10^−3^) for under fives who did not seek health care. Quality of life years loss per episode was less for older children and adults, estimated as 1.950 × 10^−3^ (0.185‐9.578 × 10^−3^) and 1.543 × 10^−3^ (0.136‐6.406 × 10^−3^) for those who seek or do not seek health care, respectively.

**Conclusion:**

Evaluations of potential RSV vaccination programmes should consider their impact across the whole population, not just young child children.

## 1 INTRODUCTION

Respiratory syncytial virus (RSV) is a leading cause of lower respiratory tract infection in infants, accounting for more than three million hospital admissions and 60 000 deaths in hospitals for children less than 5 years of age in 2015.^1^, ^2^ The health burden of RSV is high because there is no licensed vaccine for RSV, leaving infants vulnerable to infection. However, with over 40 RSV vaccine candidates currently in preclinical and clinical trials, it is likely that a vaccine will come to market in the near future.^3^ Decisions regarding the introduction of future vaccines will be informed by their projected impact and cost‐effectiveness.

Cost‐effectiveness analysis relies on the existence of measures for morbidity and mortality associated with RSV episodes routinely expressed in terms of quality of life year (QALY) loss.^4^ QALY loss for a single RSV episode is calculated by integrating the loss of health‐related quality of life (HR‐QoL) over the duration for which the symptoms are experienced. Health‐related quality of life is evaluated through the use of standardised and validated instruments, such as EuroQol's EQ‐5D that considers the physical, mental and emotional effects of an illness.^5^ Despite RSV vaccine strategy cost‐effectiveness analyses being published,^6^, ^7^ we do not have validated QALY estimates for either children or adults.^8^, ^9^, ^10^ Moreover, EQ‐5D methods do not reliably capture the HR‐QoL in very young children, in whom severe RSV episodes predominantly occur.^11^

In this study, we determined the HR‐QoL loss due to an RSV episode in individuals 5 years and older with suspected RSV using EQ‐5D questionnaires.^12^, ^13^, ^14^ We developed a statistical model to predict the HR‐QoL loss as a function of responses from a broader health and healthcare‐seeking questionnaire. We then used this statistical model to estimate QALY loss for children and adults over 5 years of age. Finally, using the statistical model parameterised with responses from the broader health and healthcare‐seeking questionnaire administered to caregivers of children aged younger than five with recently confirmed RSV infection, we calculated the QALY loss per RSV episode for children less than five.

## 2 METHODS

### 2.1 Study recruitment

During the 2016‐17 RSV season, confirmed cases of RSV in children under the age of 5 years from the previous two weeks were extracted on dates 13th December, 25th December 2016 and 3rd January 2017 from the Public Health England (PHE) Respiratory DataMart surveillance (RDMS) system.^15^ For all the confirmed cases for whom name, date of birth and National Health Service (NHS) number were provided, home addresses were obtained from the PHE Patient Demographic Service. For all these home addresses, a questionnaire pack addressed to the parent or guardian of the confirmed case was sent the day after its extraction date from the PHE RDMS system. Each questionnaire pack consisted of three questionnaires, an information sheet and a stamped addressed return envelope. The Index Questionnaire requested information about the recent RSV episode in the confirmed case. The other two questionnaires requested information about suspected RSV episodes in older household members, those aged 5‐14 years (5‐14 Questionnaire) and those aged 15 years or older (15+ Questionnaire). Suspected RSV cases were defined as persons who share a household with the confirmed case and who experienced an onset of RSV‐like symptoms (runny or blocked nose, fever, coughing, and/or a sore throat) between 5 days before and 5 days after the onset of symptoms in the confirmed case.^16^, ^17^

### 2.2 Questionnaire information

The Index Questionnaire was completed by a parent or guardian on behalf of the confirmed case, the 5‐14 Questionnaire on behalf of or by the child themselves and the 15+ Questionnaire by the adolescent or adult. The Index Questionnaire requested information on (a) the age of the child, (b) the confirmed case's symptoms (runny/blocked nose, fever, coughing, sore throat), (c) the healthcare‐seeking behaviour (no healthcare sought, contacted or visited GP, visit to Accident and Emergency department, admission to hospital), (d) coughing severity (mild/no coughing, severe coughing) and (e) a Visual Analogue Scale (VAS) for the worst day of the recent infection and the day of questionnaire completion. A VAS was presented for health from 0 (worst health) to 100 (best health) for both days and the difference between the VAS scores was defined as the VAS score loss due to an RSV episode. In addition to the questions asked in the Index Questionnaire, the 5‐14 and the 15+ Questionnaires also asked (f) the time taken off school/work due to symptoms (productivity) and (g) EuroQol EQ‐5D‐3L‐Y^12^, ^13^, ^14^ (for 5‐14 year olds)^7^ or EQ‐5D‐3L^12^, ^13^ (for 15+ year olds) questionnaires to determine HR‐QoL weight at baseline and on the worst day of suspected RSV infection. See Appendix S1 for full questionnaire packs.

The EuroQol ED‐5D‐3L‐Y^12^, ^13^, ^14^ and EQ‐5D‐3L^12^, ^13^ questionnaires use a UK‐specific time trade‐off scoring tariff to determine the HR‐QoL weight according to five dimensions: mobility, self‐care, usual activities, pain/discomfort and anxiety/depression. We refer to this HR‐QoL weight on the worst day of infection as the peak HR‐QoL weight from the data and the difference in the HR‐QoL weights between the baseline and the worst day of infection as the peak HR‐QoL loss from the data.

### 2.3 Statistical model to estimate HR‐QoL

EQ‐5D questionnaires are not validated for children under 5 years of age so we cannot obtain estimates for the peak HR‐QoL loss from the data in the confirmed cases. Therefore, using the responses from the suspected cases, we fitted a regression model to predict the (model‐estimated) peak HR‐QoL loss, as a function of questionnaire response variables: age (5‐14 years, 15 years and older), coughing severity, healthcare‐seeking behaviour, productivity and VAS score loss (Appendix S2 Section 1). To fit the regression model, we only included responses from suspected cases which, in addition to providing answers to the aforementioned response variables, completed all five dimensions of health status in their EQ‐5D questionnaires. To assess the accuracy of the fitted regression model, we compared the peak HR‐QoL loss from the response data to the model‐estimated peak HR‐QoL loss value from the fitted model. To determine the model‐estimated peak HR‐QoL loss in confirmed cases, we only included confirmed questionnaire responses which provided answers to coughing severity, productivity loss, healthcare‐seeking behaviour and VAS score loss. All analysis was performed in r (v. 3.3.2), and plotting was performed in mathematica (v. 10.3.0.0). Full details of the statistical model are provided in Appendix S2.

### 2.4 Quality‐adjusted life year (QALY) loss due to an RSV episode

We estimated each respondent's QALY loss by multiplying their model‐estimated peak HR‐QoL life loss by (a) a pooled duration of coughing distribution and (b) a fixed scaling factor for disease severity throughout the illness. We estimated the pooled coughing duration distribution separately for under fives, 5‐14 years and 15+ years, and for whether there was severe coughing (Appendix S2, Section 4). In estimating the pooled coughing duration distributions, we excluded responses that did not indicate a duration of coughing and those that indicated a duration of more than 22 days as this is longer than the maximum duration of RSV symptoms reported in previous studies.^16^ We estimated the scaling factor for disease severity using daily EQ‐5D questionnaires from individuals participating in the Flu Watch study^18^ (Appendix S2 Section 3). Due to study design, we did not collect data on HR‐QoL loss in children under the age of 5 years who did not seek health care. Therefore, to estimate the QALY loss for that group, we assumed that the ratio of QALY loss for people who seek health care to those that do not is independent of age.

### 2.5 Annual QALY loss due to RSV in England

We estimated the annual QALY loss for individuals under five and over five years old due to healthcare‐seeking RSV infections in England by multiplying our per‐episode age‐specific estimate of QALY loss with a previous estimate of the age‐specific annual number of GP consultations and hospital admissions due to RSV in England.^6^, ^19^

### 2.6 Ethics approval

In accordance with The Health Service (Control of Patient Information) Regulations 2002 No. 1438 Section 251 Regulation 3, Public Health England may process confidential patient information with a view to monitoring and managing; outbreaks of communicable disease; incidents of exposure to communicable disease and the delivery, efficacy and safety of immunisation programmes.^20^ All questionnaires that were returned from households and stored at PHE had no identifying information.

## 3 RESULTS

### 3.1 Questionnaire responses

**Figure 1:**
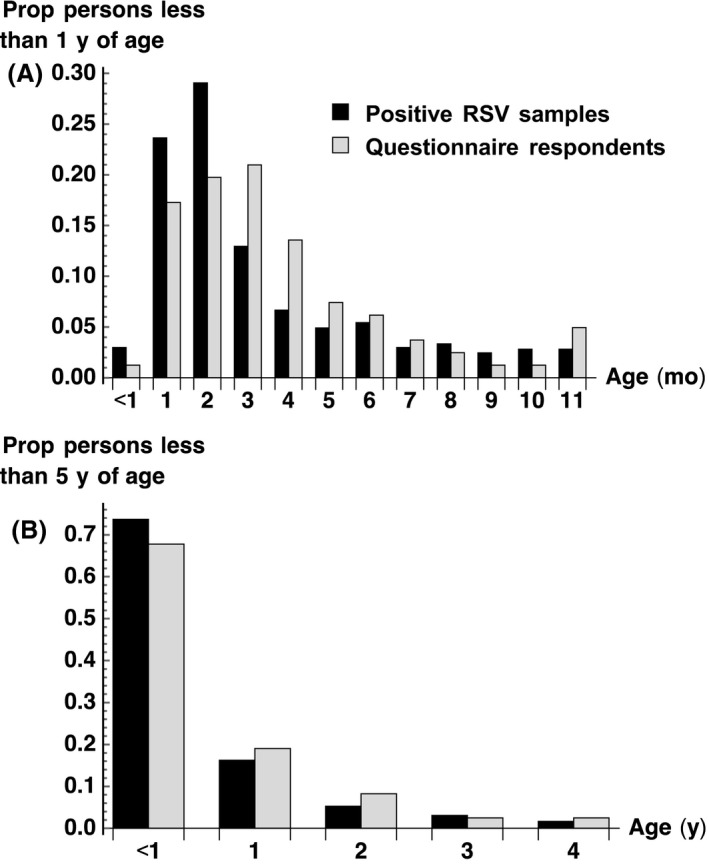
Age of confirmed RSV samples in PHE database (N = 770, black) and of the returned questionnaires for analysis (N = 122, grey)

We sent out 770 questionnaire packs between 15 December 2016 and 4 January 2017 and received 122 responses by 28 February 2017 (response rate of 16%). We found that, when stratified by year of age, the age distribution of the confirmed cases who responded was similar to the age distribution of the contacted confirmed RSV index cases. However, when stratified by month of age in the first year of life, we oversampled infants aged 3‐4 months old and undersampled infants aged 1‐2 months old (Figure 1A,B). In the 122 households, suspected cases were reported in 33 (27.0%) persons aged 5‐14 years old and 54 (44.2%) of persons aged 15 years or older.

After selecting questionnaire responses according to the inclusion criteria, we determined the model‐estimated peak HR‐QoL loss for 108/122 (88.5%) of confirmed cases in children less than 5 years of age, and for 21/33 (63.6%) and 40/54 (74.1%) of suspected cases aged 5‐14 years and 15 years and older, respectively. Duration of coughing was provided for 98/122 (80.3%) and 43/87 (49.4%) of confirmed and suspected cases, respectively.

**Figure 2:**
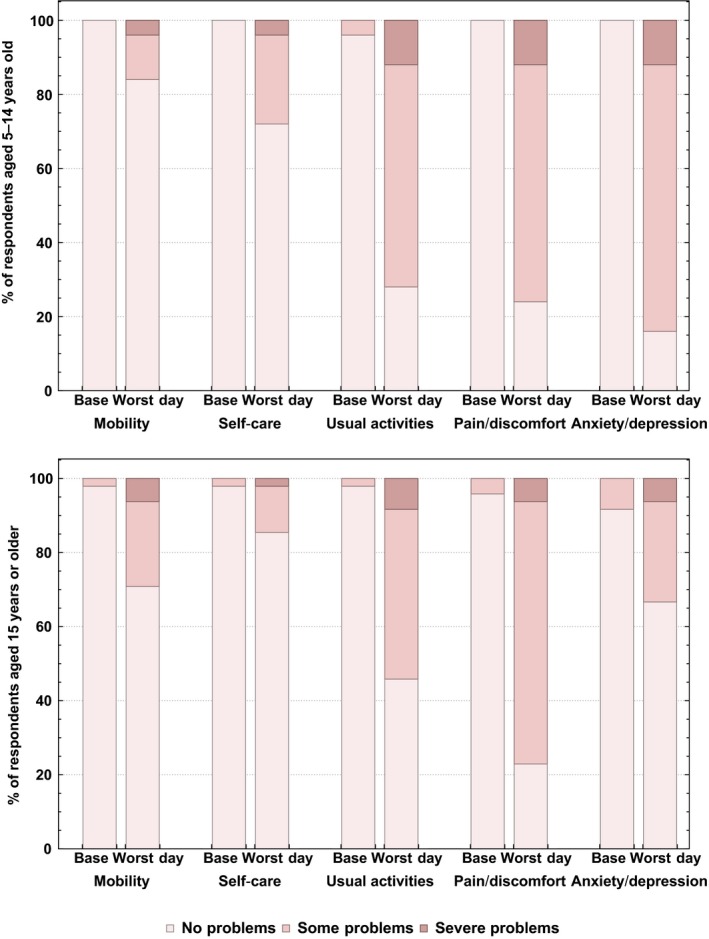
Responses from the EQ‐5D‐3L‐Y and EQ‐5D‐3L questionnaires on the day of completion (Base) and the worst day of health during a suspected infection for respondents aged 5‐14 y old (top) and 15+ y old (bottom)

In the questionnaire responses from the suspected cases, we found that 21/33 (63.6%) of children aged 5‐14 years old, and 43/54 (79.9%) of persons aged 15 years and older did not seek health care due of their suspected RSV episode (Table 1). Further, we found that 17/33 (51.5%) children aged 5‐14 years took time off school and 9/54 (16.6%) of persons aged 15 years and older took time off work or school due to their suspected RSV infection, both with a median time off of 2 days (range 1‐10 days) (Table 1). The EQ‐5D‐Y questionnaires suggested that for children aged 5‐14 years old RSV mostly affected usual activities (72%), caused pain/discomfort (76%) and anxiety/depression (84%). The EQ‐5D‐3L responses for respondents aged 15 years and older suggested similar results with RSV affecting respondents' usual activities (54.2%), causing pain/discomfort (36.0%) and anxiety and depression (32.0%) (Figure 2). Converting these EQ‐5D and EQ‐5D‐3L responses using the UK TTO scoring tariff, the median peak HR‐QoL weight from the data for children aged 5‐14 years old and persons 15 years and older was 0.689 (range −0.170‐1.000) and 0.752 (range −0.166 to 1.000), respectively (Table S2). These weights led to a median peak HR‐QoL loss from the data of 0.456 (range 0.0‐1.170) and 0.358 (range 0‐0.998) for 5‐14 and 15 years and older, respectively. As there was no significant difference between the peak HR‐QoL loss from the data between 5 and 14 years old and persons 15 years (Kolmogorov‐Smirnov test, *P* = .291), all HR‐QoL and QALY results were pooled for ages 5+ years for further analysis.

**Table 1:** Summary of index, 5‐14 and 15+ Questionnaire responses

	Aged 0‐4 y^a^ (n = 122) (%)	Aged 5‐14 y (n = 33) (%)	Aged 15 y and over (n = 54) (%)
Symptoms			
Runny/blocked nose	96 (78.7)	28 (84.8)	43 (79.6)
Fever	70 (57.4)	18 (54.5)	22 (40.7)
Coughing	110 (90.2)	27 (81.8)	51 (94.4)
Sore throat	36 (29.5)	17 (51.5)	38 (70.4)
Coughing severity			
No effect on daily activities	16 (13.1)	10 (30.3)	8 (14.8)
Mild effect on daily activities	34 (27.9)	15 (45.5)	32 (59.3)
Severe effect on daily activities	93 (76.2)	4 (12.1)	8 (14.8)
Coughing severity duration			
No effect on daily activities (median, range)	4 d (1‐14)	10 d (10‐10)	14.5 d (1‐28)
Mild effect on daily activities (median, range)	3.5 d (1‐14)	3 d (1‐9)	5.5 d (1‐28)
Severe effect on daily activities (median, range)	6.5 d (1‐35)	3 d (1‐4)	7 d (3‐10)
Healthcare‐seeking behaviour			
Phone/email NHS 111/NHS 24/NHS choices	39 (32.0)	2 (6.1)	2 (3.7)
Phone/email GP—response from the receptionist	20 (16.4)	2 (6.1)	2 (3.7)
Phone/email GP—response from the doctor or nurse	20 (16.4)	2 (6.1)	2 (3.7)
Visit a GP or nurse	83 (68.0)	10 (30.3)	10 (18.5)
Visit A&E department (including out of hours service)	71 (58.2)	3 (9.1)	1 (1.9)
Admitted to hospital	103 (84.4)	1 (3.0)	1 (1.9)
None	0 (0.0)	21 (63.6)	43 (79.6)
Productivity			
Individuals reporting taking time off work or school	—	17 (51.5)	9 (16.7)
Duration of time off work or school (median, range)	—	2 d (1‐10)	2 d (1‐7)
VAS score loss			
Baseline (median, range)	90 (30‐100)	95 (10‐100)	95 (50‐100)
Worst day (median, range)	20 (0‐85)	50 (5‐85)	50 (0‐90)
Loss (median, range)	65 (10‐100)	38 (0‐90)	35 (10‐85)

Note

Numbers in parentheses are the percentage unless otherwise stated.

Abbreviation: VAS, visual analogue scale.

a Conditional on ascertaining a confirmed case through GP/hospitalisation.

For individuals seeking health care, we found that cases in children under the age of 5 years were more severe when compared with suspected cases in persons aged 5 years and older as evidenced by a higher VAS score loss (median 65 vs 40) and the proportion of persons with severe coughing (0.76 vs 0.18).

### 3.2 Model‐estimated peak HR‐QoL loss

Using a backwards stepwise regression approach, our statistical model found that the peak HR‐QoL loss from the data was parsimoniously predicted by three factors: the VAS score loss, whether health care was sought and the presence of severe coughing (Appendix S2, Section 2 and Figures S2‐S8).

**Figure 3:**
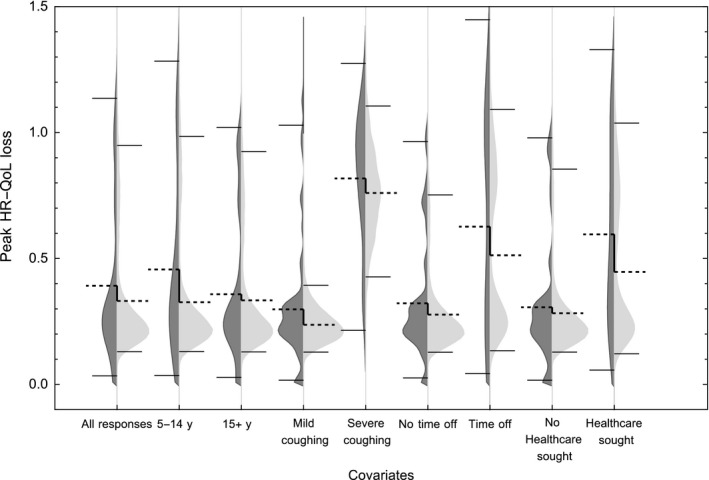
The peak HR‐QoL loss from the EQ‐5D questionnaires (dark grey) and estimated using the statistical model (light grey). The dashed line shows the mean, and solid thin lines indicate the upper and lower 95% confidence interval

Our statistical model predicted the model‐estimated peak HR‐QoL loss in suspected cases aged 5 years and older who did and did not seek health care as 0.616 (95% CI 0.155‐1.371) and 0.405 (95% CI 0.111‐1.137), respectively. We found that the questionnaire data were well predicted by the model, with no evidence to suggest the peak HR‐QoL loss from the data and the model was different (Kolmogorov‐Smirnov test, *P* = .111, Figure 3). Applying our statistical model to those under five for whom HR‐QoL loss could not be directly estimated, we found a model‐estimated peak HR‐QoL loss of 0.820 (95% CI 0.222‐1.450, Table 2; Tables S2 and S3). Finally, assuming the ratio of HR‐QoL loss for healthcare‐seeking cases to non‐health‐seeking cases is independent of age, we estimated a model‐estimated peak HR‐QoL loss for under fives as 0.539 (95% CI 0.144‐0.952, Table 2; Tables S2 and S3).

**Table 2:** HR‐QoL, QALD and QALY loss for the confirmed cases in children less than 5 y of age, and in the suspected cases in children 5 y and older

	Under 5 y of age^a^	Five years of age and older
Model‐estimated peak HR‐QoL loss (Mean and 95% CI)		
Coughing severity		
None or mild	0.499 (0.148‐1.482)	0.382 (0.111‐1.113)
Severe	0.878 (0.344‐1.443)	0.785 (0.280‐1.368)
Healthcare‐seeking behaviour		
None	0.539 (0.144‐0.952)^b^	0.405 (0.111‐1.137)
Seek healthcare	0.820 (0.222‐1.450)	0.616 (0.155‐1.371)
QALD loss (Mean and 95% CI)		
Coughing severity		
None or mild	0.845 (0.097‐3.292)	0.528 (0.050‐2.167)
Severe	1.496 (0.221‐4.841)	1.103 (0.126‐4.149)
Healthcare‐seeking behaviour		
None	1.00 (0.141‐3.652)^b^	0.565 (0.049‐2.349)
Seek health care	1.391 (0.179‐4.617)	0.866 (0.071‐3.508)
QALY loss (Mean and 95% CI)		
Coughing severity		
None or mild	2.336 × 10^−3^ (0.269‐9.255)	1.448 × 10^−3^ (0.135‐5.928)
Severe	4.098 × 10^−3^ (0.624‐13.141)	2.990 × 10^−3^ (0.346‐11.387)
Healthcare‐seeking behaviour		
None	3.024 × 10^−3^ (0.329‐10.098)^b^	1.543 × 10^−3^ (0.136‐6.406)
Seek health care	3.823 × 10^−3^ (0.492‐12.766)	1.950 × 10^−3^ (0.185‐9.578)

a Conditional on ascertaining a confirmed case through GP/hospitalisation.

b Estimated by assuming the proportional reduction in HR‐QoL loss and QALY loss between those who seek health care and those who do not is the same as observed in suspected infections in persons over 5 y of age.

### 3.3 Quality‐adjusted life years loss

The daily RSV HR‐QoL weights from Flu Watch^18^ suggest that for the first half of symptom duration, the HR‐QoL weight decreases linearly to its minimum before linearly rebounding to baseline health. There is no reported reduction of HR‐QoL weight during the second half of symptom duration. To account for the changing severity of symptoms across the entire RSV episode, we calculated the weighted HR‐QoL loss by multiplying the HR‐QoL loss by a constant scaling factor of 0.25 (Appendix S2 Section 3). Finally, to calculate the estimated QALY loss per RSV episode, we multiplied the weighted HR‐QoL loss per RSV episode by the duration of symptoms reported in our questionnaire responses (Appendix S2 Section 4). The duration of symptoms in children aged 5‐14 years was shorter (median 3 days [range 1‐10]) than both the duration of symptoms in children under 5 years old (median 5 days [range 1‐21]) and in persons aged 15 years and older (median 5 days [range 1‐21]) (Figure S10). This calculation led to an estimated QALY loss per healthcare‐seeking RSV episode in children less than 5 years old of 3.823 × 10^−3^ (95% CI 0.492‐12.766 × 10^−3^, Table 2; Tables S2 and S3)—approximately twice that for persons aged 5 years and older (1.950 × 10^−3^ (95% CI (0.185‐9.578 × 10^−3^))). For individuals who did not seek health care, the QALY loss per RSV episode was 3.024 × 10^−3^ (95% CI 0.329‐10.098 × 10^−3^) for under fives and 1.543 × 10^−3^ (95% CI 0.136‐6.406 × 10^−3^) for those 5 years and older.

### 3.4 Healthcare‐seeking and total disease burden

The total number of annual GP consultations and hospital admissions due to RSV in England is 855 000 and 375 000‐383 000 for persons aged 5 years and older and less than 5 years, respectively.^6^, ^19^ Combining these numbers with our QALY loss estimates for individuals seeking health care in England resulted in a mean annual QALY loss of 3120‐3141, 54% of which is attributable to those 5 years and older.

Our questionnaire responses indicated that 25% of individuals aged 5 years and older seek health care during an RSV episode (Table 1). Using this proportion, we estimated that there are approximately 2.6 million symptomatic RSV infections in England annually that will not be captured in a healthcare surveillance system. The mean annual QALY loss associated with these non‐healthcare‐seeking episodes for persons 5 years and older is around 4011, approximately 29% of the QALY loss in this age group.

## 4 DISCUSSION

In this study, we quantified the quality of life (QALY) loss associated with RSV episodes. For children over 5 years old and adults, we developed a statistical model that found that the QALY loss can be accurately predicted by whether there was severe coughing, whether health care was sought and Visual Analogue Scale score loss. We used our novel statistical model to evaluate the QALY loss in children under 5 years old, in whom the majority of severe RSV episodes occur but for whom QALY loss cannot be estimated directly. For those who seek health care, we found the QALY loss in children under the age of 5 years is 3.823 × 10^−3^ (95% CI 0.492‐12.766), double that for those 5 years and older (1.950 × 10^−3^ (95% CI 0.185‐9.578)). However, combining these estimates with the reported number of RSV‐associated GP consultations and hospital admissions, we find that 54% of the annual QALY burden in England is attributable to those over 5 years old.

Our study has some limitations. First, because the confirmed cases were recruited into the study conditional on them seeking health care, we could not directly estimate the QALY loss in children less than 5 years old who did not seek health care from our statistical model. To overcome this limitation, we assume that the ratio of QALY loss for people over 5 years who do not seek health care to those that do is the same independent of age. However, using this ratio may overestimate the QALY loss of non‐healthcare‐seeking cases in children less than 5 years of age, as healthcare‐seeking cases in persons aged 5 years and older are generally milder than healthcare‐seeking confirmed cases in infants less than 5 years old (with decreases in VAS score loss, coughing severity, and the proportion admitted to hospital). To collect data directly on the QALY loss in children under five who do not seek health care would require a much larger and more intensive community‐based study with frequent testing throughout an RSV season. Second, suspected cases may have experienced non‐RSV respiratory disease. However, previous studies have shown that around 50% of households experience a secondary infection in either siblings or parents during the same time as an infection in the infant; therefore, it is reasonable to assume that the majority of suspected cases are in fact RSV.^21^ Finally, completing questionnaires some days after symptoms may be subject to recall bias. Our estimates for the peak HR‐QoL life loss for persons aged 15 years and older (0.452 (95% CI 0.177‐1.222)) are larger than the peak HR‐QoL loss estimated in the Flu Watch study (range 0.107‐0.309); however, this latter estimate may be imprecise due to the small sample size.

Our study is the first to estimate the QALY loss due to acute RSV infection. Two previous studies have also estimated the HR‐QoL due to RSV infection both of which suffer from shortcomings. The first study a time trade‐off study which estimated HR‐QoL loss using responses from participants about a hypothetical illness that they, or their child, had not experienced.^8^, ^9^ Unlike this study, we calculated the HR‐QoL loss for people who have had, or suspected to have had, a recent RSV infection. The second study estimated the HR‐QoL using EQ‐5D questionnaires for children with RSV‐associated sequelae.^10^ Sequelae included chronic conditions such as persistent coughing and/or wheeze so HR‐QoL loss estimates are likely to differ substantially from those associated with acute RSV symptoms. For accurate evaluations, we recommend that future cost‐effectiveness analyses use directly obtained HR‐QoL loss estimates for RSV episodes, such as those presented in this paper, in addition to HR‐QoL loss associated with sequelae.

We present a novel statistical model to estimate QALY loss due to RSV in young children for whom standardised instruments for deriving HR‐QoL estimates are not appropriate. Thus, our method leverages the use of standardised instruments such as EQ‐5D to quantify QALY loss using more easily measurable variables of infection in young children. Our method could be applied to calculate HR‐QoL for infectious diseases for which the burden of severe disease is found in children younger than 5 years.

Our RSV‐related QALY loss estimate for people aged 5 years and over is consistent to the estimates of a prospective study that estimated QALY loss across people of all ages with non‐confirmed Influenza who reported influenza‐like illness (ILI) (mean 2.6 × 10^−3^ (range −69.2 to 39.7 × 10^−3^)).^22^ Similarly, for the under fives, our estimates are similar to non‐influenza episodes who suffer ILI who present at a hospital or GP (4.0 × 10^−3^ (range 3.4‐4.6 × 10^−3^)).^23^ In contrast, we find that our QALY loss estimates for RSV episodes in the under fives who seek health are less severe than hospitalised influenza episodes, (QALY loss of 6.0 × 10^−3^ (range 5.1‐6.9 × 10^−3^)).^23^ These comparisons suggest that, although influenza has a higher QALY loss per episode, the QALY loss due to an RSV episode is comparable to previous QALY loss estimates for persons with general ILI.

We estimated that 54% of the QALY loss associated with healthcare‐seeking episodes was attributable to individuals aged 5 years and older. This result suggests that neglecting QALY loss in older children and working‐age adults might substantially underestimate the impact of a potential RSV vaccine programme. Further, our results are consistent with previous studies that suggest that RSV is characterised by high levels of household transmission.^21^, ^24^ Together, these data suggest that integrating transmission models—that capture both the direct and indirect effects of immunisation—into economic evaluations will be crucial to accurately estimate the impact of potential vaccine programmes.

From our questionnaires, we are unable to estimate the proportion of healthcare seeking in children younger than 5 years with symptomatic RSV. However, we expect this proportion to be higher than the 25% reported in those aged 5 years and older for two reasons: infections in infants are generally more severe, with higher rates of symptomatic infections^25^ and a tendency for increased parental healthcare seeking for infants and toddlers. However, the healthcare‐seeking behaviour for both children and adults will likely depend on the country and we suggest caution in translating our total RSV burden estimates for England to other countries.

In summary, we estimated the QALY loss due to an RSV episode in confirmed cases in children less than 5 years old and suspected cases in persons aged 5 years and older. Despite severe RSV being associated with infants, we found that RSV infections in individuals aged 5 years and older account for 54% of the annual QALY loss attributable to healthcare‐seeking episodes in England. Consequently, economic evaluations of potential vaccine programmes should consider the effect on reducing incidence not only where the severe disease burden lies, but across the whole population.

## CONFLICT OF INTEREST

None declared.

## AUTHOR'S CONTRIBUTIONS

DH, KEA, MB, JPG, AT, AJvH and RP conceived and designed the surveillance study. DH, DT and HZ were involved in the data extraction, the distribution of the questionnaires and data input of the questionnaire responses. EF and AH were involved in collecting and interpreting the Flu Watch data. DH performed the statistical analysis with interpretations from KEA, MB, JPG, RP. DH, KA, RP, MB and JPG drafted the manuscript with critical revisions from DT, AJvH, HZ, EF, AH.
