# Supplementary material for: Estimates for quality of life loss due to Respiratory Syncytial Virus
Source: Influenza Other Respir Viruses. 2019 Oct 18;14(1):19–27. doi: 10.1111/irv.12686 (PMC6928035; doi:10.1111/irv.12686)
Supplement: Supplementary file 1 [file IRV-14-19-s001.docx]

**Appendix II for**

**Estimates of quality of life loss due to RSV**

Table of contents

1. Methods: Regression analysis
   1. Model selection
   2. Mixture model structure
   3. Explanatory variables
   4. Backwards stepwise regression
   5. Estimating the variance
   6. Data used to fit the mixture model
2. Results: Regression analysis

2.1 Linear regression model for severe disease

2.2 Log-transformed linear regression model for mild disease

2.3 Logistic regression model for classification of disease severity

2.4 Summary of results

1. Estimating the HR-QoL loss through duration of symptoms
2. Duration of symptoms
3. HR-QoL weight for the explanatory variables

5.1 Suspected cases aged 5 years and older

1. Model-estimated peak HR-QoL loss and QALY loss for the explanatory variables

6.1 Confirmed cases under 5 years of age

6.2 Suspected cases aged 5 years and older

This appendix gives the details of the regression model we used to determine the model-estimated peak HR-QoL loss as a function of the questionnaire response variables: age (5–14 years, 15 years and older), coughing severity, healthcare seeking behaviour, productivity and VAS score loss.

**1. Methods: Regression analysis**

**1.1. Model selection**

To inform the structure of the regression model, we first determined whether the peak HR-QoL loss sampled from the data was likely drawn from a bimodal or unimodal distribution using an F-test-based hypothesis test [1]. The variance for the bimodal distribution is calculated by dividing the data with values below and above a fixed value *h** and finding the mean of the two variances of the two groups. The degrees of freedom for an estimate of the variance of the unimodal sample is 72, and the degrees of freedom for an estimate of the variance of the bimodal sample is 69. Therefore by varying *h** within the range 0 to 1, we calculated the *h** that minimised the F-test value with 72 degrees of freedom in the numerator and 69 degrees of freedom in the denominator.

To parameterise a regression model, we use a mixture-model approach that uses three sub-models. Specifically, we estimate three response variables (a) the probability that a disease episode is severe, and the “model-estimated” peak HR-QoL loss for (b) mild ($Y_{M})$and (c) severe ($Y_{S})$ disease respectively. Therefore, the full mixture model we develop to determine the model-estimated peak HR-QoL loss for a symptomatic infection, *Y*, is given by

$${Y=pY_{S}+(1-p)Y}_{M}$$

Where *p*, $Y_{S}$, $Y_{M}$ are estimated response variables.

**1.2. Mixture model structure**

**Probability of severe disease**: To estimate the probability (*p*) of a disease episode being severe we use a logistic regression analysis because the response variable is binary (severe or mild).

**Model-estimated peak HR-QoL loss for severe and mild disease**: We initially fit $Y_{S}$ and $Y_{M}$ as linear regression models. However, in the case of $Y_{M}$linear regression resulted in unrealistic negative values. To overcome this, we used a log-transformed model instead for $Y_{M}$. For consistency, we then considered both the linear and log-transformed regression models for $Y_{S}$ and chose the better fitting model based on the Akaike Information Criterion (AIC).

Overall, for our mixture-model approach, we therefore fit three regression models: a logistic regression model to predict the probability of a disease episode being severe (*p*), a log-transformed linear model to predict the model-estimated peak HR-QoL loss when disease is mild ($Y_{M}$), and a linear model to predict the model-estimated peak HR-QoL loss when disease is severe ($Y_{S}$).

We use the R function *lm* to fit the functions:

$$E\left[ Y_{S} \right]= a_{0}+ a_{1}x_{1}+ a_{2}x_{2}+ a_{3}x_{3}+ a_{4}x_{4}+ a_{5}x_{5}$$

$$E[\log Y_{M}]=b_{0}+b_{1}x_{1}+b_{2}x_{2}+b_{3}x_{3}+b_{4}x_{4}+b_{5}x_{5}$$

and the R function *glm* with binomial family to fit the function:

$$E\left[ p \right]=\sigma\left( t \right)=\frac{1}{1+\exp\left( -t \right)},$$

$$where t= c_{0}+ c_{1}x_{1}+ c_{2}x_{2}+ c_{3}x_{3}+ c_{4}x_{4}+ c_{5}x_{5}$$

**1.3. Explanatory variables**

We used the following five explanatory variables ***x*** = (*x_1_*,…, *x_5_*) and level stratification (listed below in curly brackets). We performed the regression analyses for each of the six combinations of the explanatory variable level stratifications (three stratifications for coughing severity and two stratifications for age). The number of levels for both variables were chosen based on the AIC.

**Healthcare-seeking behaviour (**$\boldsymbol{x}_{\boldsymbol{1}}$**):** {No healthcare sought, healthcare sought}, {No healthcare sought, A+E or GP consultation but no hospital admission and admission to hospital}, {No healthcare sought, A+E but no hospital admission, GP but no hospital admission, and admission to hospital}.

**Coughing severity** $\boldsymbol{(}\boldsymbol{x}_{\boldsymbol{2}}$): {None/mild coughing, severe coughing}, {No coughing, mild coughing, and severe coughing}.

**Age**$\boldsymbol{(}\boldsymbol{x}_{\boldsymbol{3}}$): {5-14 years, 15+ years}.

**Productivity** $\boldsymbol{(}\boldsymbol{x}_{\boldsymbol{4}}$**):** { no time off work or school, time off work or school}.

**VAS score loss** $\boldsymbol{(}\boldsymbol{x}_{\boldsymbol{5}}$**):** integer value as the difference between score reported on worst day subtracted from score reported on the day of questionnaire completion.

**1.4. Backwards stepwise regression**

For each model we performed a backwards stepwise regression by estimating all five coefficients and intercept and eliminating the explanatory variable with the highest *P*-value above 0.05. We continued this process of fitting the regression model and eliminating one explanatory variable until all the remaining variables have a *P* <0.05.

**1.5 Estimating the variance**

Using the backward stepwise regression, we estimated the functions $E\left[ Y_{S} \right]$, $E\left[ \log\left( Y_{M} \right) \right]$, and $E\left[ p \right]$ as a function of the significant explanatory variables. Therefore, for an arbitrary point $\boldsymbol{x}^{\boldsymbol{*}}\boldsymbol{=\{}\boldsymbol{x}_{\boldsymbol{1}}\boldsymbol{, \ldots,}\boldsymbol{x}_{\boldsymbol{5}}\boldsymbol{\}}$**,**  the uncertainty around the estimates is given by

$$Y_{S}=\boldsymbol{A}\boldsymbol{x}^{\boldsymbol{*}}+\epsilon_{S}^{*}$$

$$\log\left( Y_{M} \right)=\boldsymbol{B}\boldsymbol{x}^{\boldsymbol{*}}+\epsilon_{M}^{*}$$

$$p=\sigma(\boldsymbol{C}\boldsymbol{x}^{\boldsymbol{*}}+\epsilon_{p}^{*})$$

Where ${\epsilon^{*}}_{j}\sim N\left( 0,\sigma_{j}^{*} \right) j\in\{S, M, p\}$ is the error associated with the prediction at point and $\mathbf{A}= \left\{ a0,\ldots,a5 \right\}, \boldsymbol{B} = \left\{ b0,\ldots,b5 \right\}, \boldsymbol{C} = \left\{ c0,\ldots,c5 \right\}$. We choose $\sigma_{S}^{*}$ and $\sigma_{M}^{*}$ to provide 95% confidence intervals that are consistent with the 95% prediction intervals from *lm* and we choose $\sigma_{p}^{*}$ to provide 95% confidence intervals that match the 95% confidence interval of the *glm* output. We take 10,000 samples from the distributions for *Y*_S_, *Y*_M_ and *p* and substitute these values into the equation for Y 10,000 times to get the empirical distributions for Y. The 95% CIs are found by ordering the data and taking the 250th and 9750th sample.

**1.6. Data used to fit the mixture model**

To fit the three regression models for *Y*_S_, *Y*_M_ and *p* we use three datasets which are derived from the observation data. These datasets consist of a response variable associated with the peak HR-QoL loss from the data and the explanatory variables, ***x*** = (*x_1_*,…, *x_5_*). For the log-transformed linear regression model, which predicts the model-estimated peak HR-QoL loss for mild disease, we use observational data for which the peak HR-QoL loss is below and equal to *h**. Similarly, for the linear regression model which predicts the model-estimated peak HR-QoL loss for severe disease, we use observational data for which the peak HR-QoL is above *h**. For the logistic regression model which predicts the probability of an infection being severe, *p*, we use all the observation data, and transform the peak HR-QoL loss from the data into a binary response variable which is 0 when the peak HR-QoL loss is below *h** and 1 when the peak HR-QoL loss is above *h**.

**2. Results: Regression analysis**

**2.1 Model selection**

The F-test-based hypothesis test value was minimised at *h**=0.6 where *F_73,69_*=4.35 and *P*<0.001, suggesting that the peak HR-QoL loss from the data were sampled from two independent distributions. Intuitively, therefore, this result is consistent with RSV disease being classed as either mild (typically with a peak HR-QoL loss from the data below the threshold value, *h**=0.6) or severe (typically with a peak HR-QoL loss from the data above the threshold value, *h**=0.6) (**Figure S1**).

**Figure S1:** Histogram of the peak HR-QoL loss due to RSV from the responses of the 5-14 and 15+ Questionnaire with the threshold value, $h^{*}$ (dashed lined), region of mild disease (green) and region of red disease (red).

**2.2. Linear regression model for severe disease**

From this linear regression we find that the single explanatory variable healthcare-seeking behaviour (stratified by 2 levels) most parsimoniously predicts the observational data for when the peak HR-QoL loss from the data is above 0.6 (**Figure S2**). The best-fit model is therefore given by $E\left[ Y_{S} \right]=0.7824+0.2436x_{1}$ (t-value = 4.111 and *P* = 0.0012, AIC = -18.6) where *x*_1_ = 0 when no healthcare is sought and 1 otherwise. The model-estimated peak HR-QoL loss for severe disease, stratified by healthcare-seeking behaviour can then be calculated (**Figure S3**).

Log-transforming the response variable reduces the likelihood (AIC = -9.7, results not shown), therefore we used an untransformed linear regression model.

**Figure S2.** Backwards stepwise regression analysis results for the five explanatory variables for the linear regression model for severe disease episodes. The *P*-value for each explanatory variable for each step in the backwards regression is shown, with an “X” indicating the variable was removed from the regression because it was the highest *P*-value above the threshold value of 0.05 (indicated by the pink region**).** The AIC value is the model-fit at the end of the regression with the remaining explanatory variables.

**Figure S3.** The distribution for the model-estimated peak HR-QoL loss for severe disease. The solid black line indicates the mean of the distribution, the dotted lines indicate lower and upper 95% CI.

**2.3. Log-transformed linear regression model for mild disease**

From this log-transformed linear regression we find that the single explanatory variable, VAS score loss, most parsimoniously predicts the observational data when the peak HR-QoL loss from the data is below 0.6. The best-fit model is therefore given by $\log\left( E\left[ Y_{M} \right] \right)=-1.7885+0.0102x_{5}$ (t-value = 4.0674, *P* = 0.000231 and AIC = 17.29) where *x*_5_ is the VAS score loss. The model-estimated peak HR-QoL loss for mild disease, for VAS score loss values between 0 and 100, can then be calculated (**Figure S5**).

**Figure S4** Backwards stepwise regression analysis results for the log-transformed linear regression model for mild disease episodes. The *P*-value for each explanatory variable for each step in the backwards regression is shown, with an “X” indicating the variable was removed from the regression because it was the highest *P*-value above the threshold value of 0.05 (indicated by the pink region.**)** The AIC value is the model-fit at the end of the regression with the remaining explanatory variables.

**Figure S5. The** distribution for the model-estimated peak HR-QoL loss for mild disease as VAS loss increases. The solid black line indicates the mean of the distribution, the dotted lines indicate lower and upper 95% CI bands.

**2.4. Logistic regression model for classification of disease severity**

From this logistic regression model, we find that coughing severity (stratified by two levels) most parsimoniously predicts the observation data when the peak HR-QoL loss is transformed to a binary response variable; 0 when less than 0.6 and 1 when greater than 0.6. The best-fit model is therefore given by $E\left[ p \right]= \sigma(-1.9924 + 3.4965x_{2})$ (*Z* = 3.908, *P* = 9.31e-05, AIC = 46.35) where *x*_2_ is 0 with no/mild coughing and 1 otherwise (**Figure S6, Figure S7**)

**Figure S6.** Backwards stepwise regression analysis results for the five explanatory variables for the logistic regression model. The *P*-value for each explanatory variable for each step in the backwards regression is shown, with an “X” indicating the variable was removed from the regression because it was the highest *P*-value above the threshold value of 0.05 (indicated by the pink region.**)** The AIC value is the model-fit at the end of the regression with the remaining explanatory variables.

**Figure S7.** The distribution for the probability of severe disease, p. The solid black line indicates the mean of the distributions, the dotted lines indicate lower and upper 95% CI.

**2.5. Summary of results**

We have used the mixture-model approach to calculate the “model-estimated” peak HR-QoL loss for a symptomatic RSV infection. We parameterized the model using questionnaire responses from respondents over five years of age with suspected RSV infection for whom we could estimate the peak HR-QoL loss derived from the EQ-5D questionnaires. We therefore determined the model-estimated peak HR-QoL loss as a function of healthcare seeking behaviour, productivity loss, age, VAS score loss, coughing severity – all of which we knew from the questionnaire respondents.

Our results suggest that the model-estimated peak HR-QoL loss due to an RSV disease episode can be predicted by clinical information independent of a validated HR-QoL questionnaire. Specifically, the model-estimated peak HR-QoL loss can be estimated using only the VAS score loss, whether healthcare was sought, and whether severe coughing was experienced (**Figure S8**).

**Figure S8.** The distribution of the model-estimated peak HR-QoL for coughing severity, healthcare-seeking behaviour and VAS loss. The solid red line indicates the mean of the distribution, the black lines indicate lower and upper 95% CI bands.

**3. Estimating the HR-QoL loss through duration of symptoms**

Flu Watch is a community cohort study in which householders were asked to prospectively record all respiratory illnesses and submit a nasal swab for Polymerase Chain Reaction (PCR) based identification of respiratory viruses over winter seasons [2]. In 2010/11 participants (or adult carers) were also asked to complete a one-off baseline EQ-5D questionnaire at the start of the study as well as daily EQ-5D questionnaires throughout any respiratory illness to measure their daily HR-QoL. We calculated the HR-QoL loss throughout an RSV episode relative to the worst day of infection for five of the nine confirmed infections. Three people were excluded as they indicated no HR-QoL loss over their infection; and one person was excluded because their base HR-QoL was lower than during the RSV episode—leading to negative HR-QoL loss values. The remaining five patients were aged 16–45 years. Their daily HR-QoL weight measures indicate that for the first half of symptom duration, the HR-QoL weight decreases linearly to its minimum before linearly rebounding to baseline health. There is no reported reduction of HR-QoL weight during the second half of symptom duration. To account for the changing severity of symptoms across the entire RSV episode, we calculated the weighted HR-QoL loss by multiplying the model-estimated peak HR-QoL loss by a constant scaling factor of 0.25. Therefore, the resulting formula for the QALY loss due to an RSV infection, Q, is Q = H*D*0.25, where H is model-estimated peak HR-QoL loss, and D is the duration of infection.

**4. Duration of symptoms**

Due to a poor response rate for reporting the duration of symptoms in our questionnaire (67%), calculating the QALY loss only for the responses which provided a duration of symptoms would lead to a substantial waste of data. Therefore, we pooled the responses for the duration of symptoms. Thus, to evaluate the QALY loss for each model-estimated peak HR-QoL loss, we then sampled a duration from this pooled distribution.

To compare using one pooled distribution to multiple distributions, stratified by respondent characteristics, we performed a backwards stepwise linear regression analysis using the duration of symptoms as the response variable and age group (<5 ,5–14, 15+ years), coughing severity (severe coughing or no/mild coughing), and VAS score loss as independent variables. We found that VAS score loss was unlikely to account for any of the variance in duration symptoms (*P* = 0.53), we pooled responses by age and coughing severity (*P* = 0.14, *P* = 0.08, respectively). Therefore, we calculated six pooled distributions for the duration of symptoms (**Figure S9**). For each included individual respondent in the analysis, we then randomly sampled their respective symptom duration from their respective pooled distribution based on their age group and whether they reported severe coughing.

**Figure S9.** The distribution of duration of coughing symptoms for all respondents with no severe coughing (upper panel) and with severe coughing (lower panel).

**5. HR-QoL weight for the explanatory variables**

**5.1 Suspected cases aged 5 years and older**

|  | **HR-QoL weight (Median and range)** |
| --- | --- |
| **Age (years)** | |
| 5–14 | 0.689 (-0.170–1.000) |
| 15+ | 0.752 (-0.166–1.000) |
| **Coughing severity** | |
| None or mild | 0.760 (-0.126–1.000) |
| Severe | -0.008 (-0.170–0.691) |
| **Healthcare-seeking behaviour** | |
| None | 0.743 (-0.077–1.000) |
| Seek healthcare | 0.300 (-0.170–1.000) |
| **Productivity** | |
| Time taken off | 0.760 (-0.166–1.000) |
| No time taken off | 0.439 (-0.170–0.812) |
| **VAS score loss** | |
| Below median (40) | 0.796 (-0.170–1.000) |
| Above median (40) | 0.552 (-0.166–0.812) |

**Table S1** HR-QoL weight for each explanatory variable for suspected cases in persons aged five years and older.

**6. Model-estimated peak HR-QoL loss and QALY loss for the explanatory variables**

**6.1 Confirmed cases under 5 years of age***

|  | **Model-estimated peak HR-QoL loss (Mean and 95% CI)** | **QALD loss (Mean and 95% CI)** | **QALY loss (Mean and 95% CI)** |
| --- | --- | --- | --- |
| **Age (months)** | | | |
| 0–5 | 0.798 (0.208–1.462) | 1.356 (0.161–4.643) | 3.731 × 10^-3^  (0.456–12.710) |
| 6–11 | 0.840 (0.235–1.438) | 1.429 (0.187–4.731) | 3.935 × 10^-3^ (0.505–12.889) |
| 12–23 | 0.861 (0.301–1.446) | 1.464 (0.219–4.765) | 4.043 × 10^-3^  (0.585–13.325) |
| 24–59 | 0.836 (0.244–1.419) | 1.421 (0.199–4.626) | 3.871 × 10^-3^  (0.521–12.727) |
| **Coughing severity** | | | |
| None or mild | 0.499 (0.148–1.482) | 0.845 (0.097–3.292) | 2.336 × 10^-3^  (0.269–9.255) |
| Severe | 0.878 (0.344–1.443) | 1.496 (0.227–4.841) | 4.098 × 10^-3^  (0.624–13.141) |
| **Healthcare-seeking behaviour** | | | |
| None | *No response* | *No response* | *No response* |
| Seek healthcare | 0.820 (0.222–1.450) | 1.391 (0.179–4.617) | 3.823 × 10^-3^  (0.492–12.766) |
| **VAS loss** | | | |
| Below median (65) | 0.784 (0.200–1.393) | 1.339 (0.166–4.488) | 3.676 × 10^-3^  (0.453–12.250) |
| Above median (65) | 0.860 (0.259–1.498) | 1.462 (0.202–4.865) | 3.989 × 10^-3^  (0.525–13.212) |

**Table S2.** HR-QoL, QALD and QALY loss for each explanatory variable for confirmed cases in children under the age of five.

*Conditional on ascertaining a confirmed

**6.2 Suspected cases aged 5 years and older**

|  | **Model-estimated peak HR-QoL loss (Mean and 95% CI)** | **QALD loss (Mean and 95% CI)** | **QALY loss (Mean and 95% CI)** |
| --- | --- | --- | --- |
| **Age (years)** | | | |
| 5–14 | 0.462 (0.118–1.308) | 0.637 (0.054 –2.609) | 1.740 × 10^-3^  (0.144–7.277) |
| 15+ | 0.452 (0.117–1.222) | 0.625 (0.052–2.607) | 1.717 × 10^-3^  (0.144–7.078) |
| **Coughing severity** | | | |
| None or mild | 0.382 (0.111–1.113) | 0.528 (0.050–2.167) | 1.448 × 10^-3^  (0.135–5.928) |
| Severe | 0.785 (0.280–1.368) | 1.103 (0.126–4.149) | 2.990 × 10^-3^  (0.346–11.387) |
| **Healthcare-seeking behaviour** | | | |
| None | 0.405 (0.111–1.137) | 0.565 (0.049–2.349) | 1.543 × 10^-3^  (0.136–6.406) |
| Seek healthcare | 0.616 (0.155–1.371) | 0.866 (0.071–3.508) | 1.950 × 10^-3^  (0.185–9.578) |
| **Productivity** | | | |
| Time taken off | 0.404 (0.111–1.176) | 0.558 (0.048–2.342) | 1.539 × 10^-3^  (0.137–6.382) |
| No time taken off | 0.579 (0.139–1.369) | 0.788 (0.067–3.184) | 2.170 × 10^-3^  (0.173–8.818) |
| **VAS loss** | | | |
| Below median (40) | 0.373 (0.107–1.081) | 0.524 (0.047–2.190) | 1.417 × 10^-3^  (0.131–5.749) |
| Above median (40) | 0.562 (0.141–1.382) | 0.790 (0.068–3.246) | 2.163 × 10^-3^  (0.182–8.945) |

**Table S3.** HR-QoL, QALD and QALY loss for each explanatory variable for suspected cases in persons aged five years and older.

**References**

[1] Larkin RP (1979) An algorithm for assessing bimodality vs. unimodality in a univariate distribution. Behavior Research Methods 11 (4) 467-468 DOI: 10.3758/BF03205709

[2] Fragaszy EB, Warren-Gash C, White PJ, et al. Effects of seasonal and pandemic influenza on health-related quality of life, work and school absence in England: results from the Flu Watch cohort study. Influenza Other Respi Viruses 2018 Jan; 12(1): 171–182. DOI:10.1111/irv.12506.
